# Albumin-Coated Copper Oxide Nanoparticles for Radiosensitization of Human Glioblastoma Cells Under Clinically Relevant X-Ray Irradiation

**DOI:** 10.3390/nano15171376

**Published:** 2025-09-05

**Authors:** Chanyatip Suwannasing, Nittiya Suwannasom, Pattawat Iamcharoen, Rachan Dokkham, Panupong Maun, Pitchayuth Srisai, Hans Bäumler, Ausanai Prapan

**Affiliations:** 1 Department of Radiological Technology, Faculty of Allied Health Sciences, Naresuan University, Mueang District, Phitsanulok 65000, Thailand; sawitreesu@nu.ac.th (C.S.); pattawati65@nu.ac.th (P.I.); rachand65@nu.ac.th (R.D.); panupongm65@nu.ac.th (P.M.); 2 Division of Biochemistry, School of Medical Sciences, University of Phayao, Phayao 56000, Thailand; nittiya.su@up.ac.th; 3 Department of Biology, Faculty of Science, Chiang Mai University, Chiang Mai 50200, Thailand; pitchayuth_sri@cmu.ac.th; 4 Institute of Transfusion Medicine, Charité-Universitätsmedizin Berlin, 10117 Berlin, Germany; hans.baeumler@charite.de; 5 Department of Pharmaceutical Technology and Biotechnology, Faculty of Pharmacy, Payap University, Chiang Mai, Mueang District, Chiang Mai 50000, Thailand

**Keywords:** glioblastoma, bovine serum albumin, copper oxide nanoparticles, radiosensitivity, apoptosis

## Abstract

Glioblastoma (GBM) is the most aggressive and treatment-resistant primary brain tumor in adults. Despite current multimodal therapies, including surgery, radiation, and temozolomide chemotherapy, patient outcomes remain poor. Enhancing tumor radiosensitivity through biocompatible nanomaterials could provide a promising integrative strategy for improving therapeutic effectiveness. This study aims to evaluate the potential of bovine serum albumin-coated copper oxide nanoparticles (BSA@CuO-NPs) to enhance radiosensitivity in U87-MG cells under clinically relevant X-ray irradiation. In brief, BSA@CuO-NPs were synthesized via carbodiimide crosslinking and characterized by DLS, SEM, and zeta potential analysis. U87-MG cells were treated with BSA@CuO-NPs alone or in combination with X-ray irradiation (2 Gy). Cytotoxicity was assessed using the MTT assay, while radiosensitization was evaluated through clonogenic survival analysis. Apoptosis induction and DNA damage were analyzed via Annexin V staining and γ-H2AX immunofluorescence, respectively. The results revealed that BSA@CuO-NPs showed good colloidal stability and biocompatibility compared with uncoated CuO-NPs. When combined with irradiation, BSA@CuO-NPs significantly decreased clonogenic survival (*p* < 0.05) and increased apoptotic cell death compared to irradiation alone. Immunofluorescence demonstrated increased γ-H2AX focus formation, indicating higher DNA double-strand breaks in the combination group. In conclusion, BSA@CuO-NPs enhance the effects of ionizing radiation by increasing DNA damage and apoptosis in U87-MG cells, indicating their potential as combined radiosensitizers. These results support further research into albumin-coated metal oxide nanoparticles as adjuncts to standard radiotherapy for the management of GBM. One challenge in this context is the effective delivery of nanoparticles to GBM. However, the stability of BSA@CuO-NPs in physiological solutions could help overcome this obstacle.

## 1. Introduction

Glioblastoma (GBM) represents the most aggressive and treatment-resistant primary brain tumor in adults, constituting approximately 48% of all malignant tumors of the central nervous system [1,2]. Despite aggressive multimodal strategies, including maximal surgical resection, adjuvant radiotherapy, and concomitant as well as adjuvant temozolomide (TMZ) chemotherapy, the prognosis for GBM remains poor, with a median survival of around 12–15 months [3]. A significant obstacle to effective treatment is the natural radioresistance of GBM cells, which results from several factors, including efficient DNA repair, a hypoxic tumor environment, and defective apoptosis signaling.

Radiotherapy (RT) remains a cornerstone in the treatment of GBM, yet its efficacy is frequently hindered by the intrinsic resistance of the tumor to ionizing radiation. This resistance is primarily attributed to the ability of GBM cells to efficiently repair sublethal DNA damage and evade radiation-induced mitotic catastrophe [4,5]. As a result, enhancing the radiosensitivity of GBM cells has become a central focus in the development of adjunctive therapeutic strategies, among which the use of nanoparticles as radiosensitizers has gained increasing attention [6]. Nanoparticles composed of high-atomic-number (Z) elements can amplify local radiation effects by increasing photoelectric and Compton interactions, thereby promoting localized energy deposition and the generation of reactive oxygen species (ROS) [7,8]. These ROS, such as hydroxyl radicals and superoxide anions, induce oxidative stress, mitochondrial dysfunction, and extensive DNA damage, especially double-strand breaks (DSBs), which represent the most lethal form of radiation-induced injury [9]. Metal oxide nanoparticles, due to their unique physicochemical properties like enhanced reactivity due to high surface area, tunable bandgap, ROS generation, shape control, and redox activity due to Cu^2+^ ↔ Cu^+^ cycling, represent a promising subclass of nanomaterials that can enhance both the physical and biological effects of radiation, thereby offering new opportunities for improving GBM radiotherapy outcomes [7,10,11].

Beyond their role in increasing physical radiation dose, metal oxide nanoparticles have demonstrated promising biological radiosensitizing effects. Nanoparticles, such as zinc oxide (ZnO), cerium oxide (CeO_2_), and titanium dioxide (TiO_2_), are known to regulate oxidative stress, disrupt DNA repair processes, and induce apoptosis in cancer cells, thereby enhancing their sensitivity to radiation [12]. These nanoparticles can also selectively accumulate in tumor tissues through the enhanced permeability and retention (EPR) effect, thereby reducing damage to healthy tissue during radiotherapy [13]. Among them, CuO nanoparticles (CuO-NPs) are especially notable because of their atomic number (Z = 29) and strong oxidative potential. Moreover, copper-based nanomaterials have been extensively studied in biomedicine, not only for their radiosensitizing potential but also for their broad applications in antibacterial therapy, wound healing, and anticancer strategies, including the development of copper complexes [14,15,16]. In particular, CuO-NPs can increase radiation-induced cytotoxicity by producing reactive oxygen species (ROS) both independently and in combination with other factors, such as inhibition of DNA repair pathways and induction of cell cycle arrest in radiosensitive phases [6,17]. The resulting oxidative stress causes damage to cellular components, reduces mitochondrial function, disrupts the cell cycle, and increases DNA double-strand breaks, indicating a promising factor in radiosensitization [18].

However, a significant challenge in using nanoparticles clinically is their tendency to aggregate in physiological environments, reducing their bioavailability and therapeutic effectiveness. To overcome this, surface functionalization with biocompatible molecules has been examined to enhance dispersion, cellular uptake, and overall efficacy in cancer treatments [19]. In this study, surface modification with biocompatible proteins, such as bovine serum albumin (BSA), has been employed. BSA not only stabilizes nanoparticles in biological fluids but also promotes cellular uptake by forming a biocompatible protein corona that facilitates receptor-mediated endocytosis, while simultaneously reducing aggregation and surface charge repulsion. In addition, BSA coating helps extend systemic circulation by enhancing nanoparticle stability and reducing clearance via the mononuclear phagocyte system [20]. Moreover, BSA has functional groups that can be linked to nanoparticle surfaces, facilitating controlled dispersion and enhanced interaction with cancer cells [21,22,23]. BSA-coated CuO nanoparticles (BSA@CuO-NPs) thus represent an attractive nanosystem for radiosensitization, combining enhanced stability with biological compatibility.

Given their capacity to enhance reactive oxygen species, regulate the cell cycle, and suppress DNA repair mechanisms, CuO-NPs have attracted attention as radiosensitizers in GBM [6]. However, their clinical translation is hindered by poor biocompatibility, non-specific biodistribution, and instability in physiological environments. In this study, we address these challenges by synthesizing BSA@CuO-NPs and showing their capacity to increase the radiosensitivity of U87-MG glioblastoma cells during megavoltage X-ray irradiation. A comprehensive biological evaluation, including cytotoxicity, clonogenic survival, apoptosis, cell cycle arrest, and γ-H2AX-based DNA damage analysis, demonstrates their potential as a next-generation nanomedicine-based radiosensitizer and provides a foundation for future translational studies in GBM.

## 2. Materials and Methods

### 2.1. Preparation of BSA-Coated CuO Nanoparticles (BSA@CuO-NPs)

BSA@CuO-NPs were synthesized using carbodiimide crosslinking chemistry for biomedical applications, as previously reported [24]. First, 3 mg of CuO nanoparticles (Sigma-Aldrich, St. Louis, MO, USA) were dispersed in 750 µL of phosphate-buffered saline (PBS, pH 7.4) and sonicated using a sonication probe for 60 s to achieve a uniform dispersion. Separately, 5 mg each of N-hydroxysuccinimide (NHS, Sigma-Aldrich, St. Louis, MO, USA) and 1-ethyl-3-(3-dimethylaminopropyl) carbodiimide hydrochloride (EDC, Sigma-Aldrich), along with 7.5 mg of bovine serum albumin (BSA; Fraction V, Sigma-Aldrich, St. Louis, MO, USA), were dissolved in 750 µL of PBS and preconditioned in a sonication bath for 15 min. The EDC, NHS, and BSA solutions were then combined sequentially and mixed with the CuO suspension to create a total reaction volume of 3 mL. The final concentration of BSA in the reaction mixture was 2.5 mg/mL, which falls within the commonly used range for protein functionalization of metal oxide nanoparticles, ensuring effective surface coating and colloidal stability without excess unbound protein. The complete mixture was continuously stirred at room temperature using a magnetic stirrer for 24 h to facilitate the covalent conjugation of BSA to the nanoparticle surface. After incubation, the nanoparticle suspension was centrifuged at 6000 rpm for 10 min and washed twice with PBS to remove residual reagents and unbound BSA. The resulting pellet of BSA@CuO-NPs was lyophilized and stored at 4 °C until further use.

### 2.2. Characterization

#### 2.2.1. Hydrodynamic Size and Zeta Potential

The hydrodynamic diameter, PDI, and surface charge (zeta potential) of CuO and BSA@CuO nanoparticles were measured using a Zetasizer Ultra (Malvern Instruments Ltd., Malvern, UK). Bare CuO nanoparticles were initially resuspended in phosphate-buffered saline (PBS), which caused extensive aggregation in suspension. For each measurement, 2 µL of nanoparticle suspension was diluted in 1 mL of deionized water, gently mixed, and loaded into a disposable polystyrene cuvette. Measurements were performed at 25 °C after a brief equilibration period. DLS at a scattering angle of 90° was used to determine hydrodynamic diameter and PDI, while zeta potential was measured using a folded capillary cell (DTS1070) under identical dilution conditions. All measurements were performed in triplicate. The Z-average diameter, polydispersity index (PDI), and zeta potential were calculated using the instrument’s software program to evaluate the results of the obtained nanoparticles.

#### 2.2.2. Scanning Electron Microscopy (SEM) and Energy-Dispersive X-Ray (EDX) Spectroscopy

The morphology and particle size of BSA@CuO-NPs were examined using field-emission scanning electron microscopy (FE-SEM; Apreo S, Thermo Fisher Scientific, Waltham, MA, USA) at 20 kV acceleration voltage. A drop of the nanoparticle suspension was placed on a clean, conductive metal stub and air-dried at room temperature before imaging. The elemental composition was analyzed using energy-dispersive X-ray spectroscopy (EDX), integrated into the FE-SEM system. EDX spectra were collected from various areas on the sample surface to evaluate the uniformity and reproducibility of the elemental distribution.

#### 2.2.3. Fourier-Transform Infrared (FTIR) Spectroscopy

FTIR spectroscopy verified the surface modification of CuO-NPs with BSA. Spectra of bare CuO NPs, pure BSA, and BSA@CuO-NPs were recorded using a Nicolet iS5 FT-IR spectrometer (Thermo Scientific, Waltham, MA, USA). Each sample was prepared as a potassium bromide (KBr) pellet and scanned from 400 to 4000 cm^−1^ at a resolution of 4 cm^−1^ with 64 scans per spectrum. Characteristic absorption bands related to amide I and II (from BSA) and metal–oxygen bonds (from CuO) were observed, confirming the successful coating of protein on the nanoparticle surface.

### 2.3. Cell Culture Conditions and Treatment Groups

The human glioblastoma (U87-MG) cells (U87-MG cells; ATCC^®^ HTB-14™, Manassas, VA, USA) were routinely cultured in Dulbecco’s Modified Eagle Medium (DMEM; Life Technologies, NY, USA) supplemented with 10% fetal bovine serum (FBS) and 1% penicillin–streptomycin solution (HyClone Laboratories, Logan, UT, USA). Cells were incubated at 37 °C in a humidified atmosphere containing 5% CO_2_ and 95% air. The culture medium was refreshed every 2–3 days, and cells were subcultured when they reached approximately 80–90% confluence, thereby maintaining optimal growth conditions.

In the treatment experiments, U87-MG cells were divided into four groups: (1) control—cells that received no treatment; (2) BSA@CuO-NPs only—cells treated with the IC20 concentration of BSA@CuO-NPs for 24 h; (3) irradiation only—cells exposed to a single dose of 2 Gy radiation; (4) combination treatment—cells pretreated with the IC20 concentration of BSA@CuO-NPs for 24 h, followed by 2 Gy of irradiation. All treatments were performed in triplicate to ensure reproducibility and statistical validity integrity. The IC20 concentration was selected rather than IC50 to minimize inherent nanoparticle cytotoxicity and to maintain sufficient viable cells for irradiation, thereby enabling accurate evaluation of radiosensitization effects. Similar studies on nanoparticle radiosensitization have also employed sublethal concentrations, such as IC10 and IC20, for this purpose [12,25].

### 2.4. MTT Assay and IC20 Determination

The cytotoxic effects of BSA@CuO-NPs were evaluated in U87-MG cells using the MTT assay. Cells were seeded in 96-well plates at a density of 1 × 104 cells per well in 100 µL of complete DMEM and incubated at 37 °C in a humidified atmosphere containing 5% CO_2_ for 24 h to facilitate cell attachment. After incubation, cells were treated with increasing concentrations of BSA@CuO-NPs (0, 2, 4, 6, 8, 16, and 32 µg/mL) for 24 h. Subsequently, 100 μL of MTT reagent (0.5 mg/mL in incomplete DMEM; Sigma-Aldrich, St. Louis, MO, USA) was added to each well and incubated for an additional 3 h at 37 °C. Formazan crystals formed by metabolically active cells were solubilized using 100 µL of dimethyl sulfoxide (DMSO; AR grade, 99% purity, Loba Chemie, Mumbai, India), and absorbance was measured at 570 nm using a microplate spectrophotometer (BioTek Instruments, Winooski, VT, USA). All experiments were performed in triplicate. The percentage of cell viability was calculated relative to untreated control wells. The inhibitory concentration at which 20% reduction in cell viability was observed (IC20) was determined via nonlinear regression analysis using the GraphPad Prism software program (version 10). The IC20 concentration was used for subsequent radiosensitization experiments.

### 2.5. Irradiation Setup

Irradiation was performed using a 6 MV photon beam from a clinical linear accelerator (Varian Clinac 2100C; Varian Medical Systems, Palo Alto, CA, USA). U87-MG cells were seeded in 24-well plates and exposed to a single 2 Gy radiation dose. The plates were positioned horizontally at the isocenter within a tissue-equivalent bolus phantom, with an additional 1.5 cm bolus on top. The cell monolayer was placed at a depth of 3.5 cm to ensure dose buildup and electronic equilibrium. The source-to-surface distance (SSD) was maintained at 96.5 cm. A 20 × 15 cm^2^ field size was used with a vertical photon beam to deliver a uniform dose across the samples.

### 2.6. Clonogenic Survival Assay

The impact of BSA@CuO-NPs and ionizing radiation on the clonogenic survival of U87-MG cells was evaluated using a colony formation assay. In brief, 2 × 10^5^ U87-MG cells were seeded into 24-well plates and allowed to adhere overnight. Cells were then treated with BSA@CuO-NPs at the IC20 concentration for 24 h, followed by exposure to 2 Gy of X-ray radiation. Post-treatment, cells were collected, counted, and replated into 6-well plates at different densities based on expected cytotoxic effects: 300 cells/well for untreated controls, 600 cells/well for radiation-only treatment, and 1000 cells/well for the combined treatment. Cultures were maintained in a humidified incubator at 37 °C with 5% CO_2_ for 10–14 days to facilitate colony growth. Colonies were then fixed with methanol for 30 min, stained with 0.5% crystal violet (Sigma-Aldrich, St. Louis, MO, USA) for 30 min, and rinsed with distilled water. Colony counting was performed using the Colony Counter plugin (version 0.9)) in ImageJ (version 1.54g, Bruno Vieira, University of Lisbon, Lisbon, Portugal), and the results were analyzed using the ImageJ software program (National Institutes of Health, Bethesda, MD, USA) [26]. Plating efficiency (PE) was calculated as the percentage of colonies formed in the untreated control group relative to the number of cells seeded. The surviving fraction (SF) for each group was derived by normalizing the colony count to the plating efficiency of the control.

### 2.7. γ-H2AX Assay for DNA Damage Assessment

DNA double-strand breaks (DSBs) were assessed using γ-H2AX immunofluorescence staining. U87-MG cells were cultured on sterile coverslips in 24-well plates and treated with BSA@CuO-NPs at their IC20 concentration for 24 h, with untreated cells serving as controls. After treatment, the cells were exposed to 2 Gy of X-ray irradiation, washed with PBS, and fixed using 4% paraformaldehyde. They were then permeabilized with 0.1% Triton X-100 and blocked with 2% BSA. U87-MG cells were incubated overnight at 4 °C with (1:800; Thermo Fisher Scientific, Rockford, IL, USA), followed by an Alexa Fluor^®^ 488-conjugated goat anti-mouse IgG (1:1000; Life Technologies, Eugene, OR, USA) for 1 h. Nuclei were stained with DAPI, and slides were mounted with antifade medium. Fluorescence images were captured at 40× magnification using a Zeiss microscope. For each sample, three random fields were imaged. Two independent, blind observers manually counted the total nuclei and γ-H2AX-positive cells. The percentage of γ-H2AX-positive cells was averaged across the three fields to compare DNA damage between groups.

### 2.8. Apoptosis and Cell Cycle Analysis

U87-MG cells were seeded into 12-well plates and treated with BSA@CuO-NPs at the IC20 concentration for 24 h. Subsequently, the cells were exposed to 2 Gy of X-ray irradiation. Untreated cells served as the control group. After treatment, the cells were trypsinized, harvested, and washed twice with PBS before being transferred to 1.5 mL microcentrifuge tubes. For apoptosis analysis, 100 µL of the cell suspension (5 × 10^5^ cells/mL) was mixed with 100 µL of Muse™ Annexin V & Dead Cell Reagent and incubated for 20 min at room temperature in the dark. Apoptotic populations were analyzed using a Muse™ Cell Analyzer (Merck, Darmstadt, Germany), with discrimination based on Annexin V–FITC staining profiles. For cell cycle analysis, the remaining cells were fixed in 1 mL of cold 70% ethanol and incubated at 4 °C for 5 h. After fixation, 200 µL of the cell suspension (1 × 106 cells/mL) was washed with 250 µL of PBS and stained with 200 µL of Muse™ Cell Cycle Reagent. The samples were incubated at room temperature in the dark for 20 min prior to analysis using the Muse™ Cell Analyzer.

### 2.9. Statistical Analysis

All results are presented as mean ± standard error of mean (SEM), calculated from three independent experiments. Statistical comparisons were performed using an unpaired two-tailed Student’s T-test (for comparisons between two groups) or one-way and two-way ANOVA (for multiple group comparisons), followed by Tukey’s post hoc test when appropriate. All statistical analyses and graphical representations were performed using GraphPad Prism version 11. A *p*-value of less than 0.05 was considered statistically significant.

## 3. Results

### 3.1. Results of Synthesis and Characterization of BSA@CuO-NPs

BSA@CuO-NPs were successfully synthesized via carbodiimide crosslinking chemistry using EDC/NHS-mediated activation in aqueous conditions. This approach enabled coupling proteins to metal oxide surfaces by forming amide linkages between activated surface groups and protein amine [27]. In addition, spectroscopic studies have shown that BSA can also interact with CuO nanoparticles through non-covalent electrostatic and hydrogen-bonding interactions [28]. The resulting nanostructure is thus likely stabilized by both covalent (amide) and non-covalent interactions, yielding a robust protein-functionalized CuO nanoparticle.

**Figure 1:**
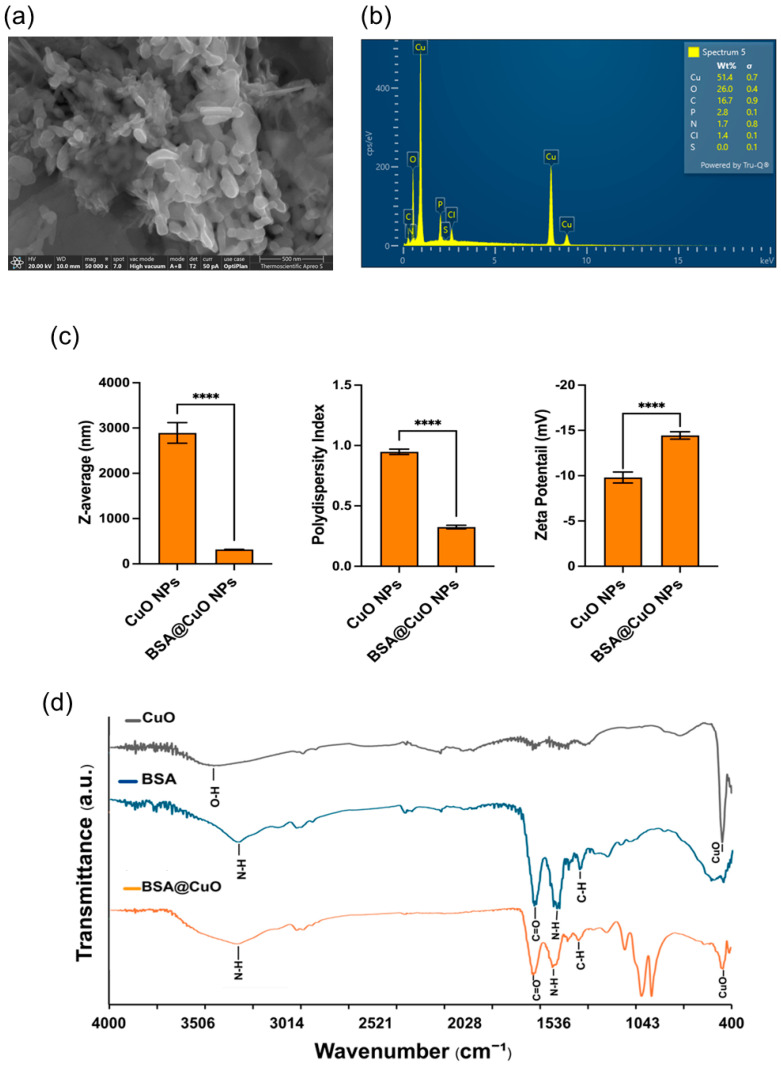
Characterization of BSA@CuO-NPs. (**a**) SEM image showing quasi-spherical particles at nanoscale size (scale bar: 500 nm). (**b**) EDS analysis confirms the presence of Cu, O, C, P, N, Cl, and S, indicating successful BSA coating. (**c**) DLS data indicate that BSA coating significantly reduces the Z-average size and PDI of CuO-NPs and slightly shifts the zeta potential, demonstrating improved dispersion and colloidal stability mainly due to the steric effects of adsorbed albumin molecules. Data are mean ± SEM (**** *p* < 0.0001). (**d**) FTIR spectra of uncoated CuO, BSA, and BSA@CuO-NPs confirm BSA functionalization.

As shown in Figure 1a, SEM imaging revealed moderately clustered, quasi-spherical nanoparticles with nanoscale sizes, consistent with the expected shape of CuO-NPs coated with BSA. The surface appeared irregular and organic, indicating successful modification with BSA. EDS analysis, presented in Figure 1b, confirmed the elemental composition of the synthesized nanoparticles. The representative spectra showed the presence of copper (Cu, 51.4 wt%), oxygen (O, 26.0 wt%), carbon (C, 16.7 wt%), phosphorus (P, 2.8 wt%), nitrogen (N, 1.7 wt%), and trace amounts of chlorine (Cl, 1.4 wt%). The detection of nitrogen and phosphorus, which are elements characteristic of the BSA peptide backbone and phosphate groups, respectively, provides strong evidence for the effective immobilization of protein on the CuO surface. These elements were notably absent in the EDS spectra of bare CuO-NPs, thereby reinforcing the successful conjugation of BSA. To provide a direct comparison, the SEM image and EDS spectrum of bare CuO-NPs are shown in the Supplementary Materials (Figure S1). Bare CuO-NPs exhibited a more irregular morphology with heterogeneous surface features and lacked characteristic protein-associated elemental signals, emphasizing the difference between uncoated and BSA-coated nanoparticles.

DLS measurements (Figure 1c) confirmed a marked improvement in colloidal stability after BSA coating. Bare CuO nanoparticles exhibited an apparent hydrodynamic diameter of approximately 3000 nm, accompanied by a very high PDI of around 1.0, which suggests extensive aggregation in the PBS suspension rather than the intrinsic nanoscale size of the particles. In contrast, BSA@CuO-NPs synthesized with 2.5 mg/mL BSA exhibited a reduced hydrodynamic size of approximately 300 nm and a significantly lower PDI (about 0.25), indicating a more uniform and well-dispersed suspension. The surface charge also shifted from approximately −10 mV for bare CuO to −15 mV for BSA@CuO-NPs, consistent with the negatively charged BSA corona and enhanced electrostatic stabilization. All changes in size, PDI, and zeta potential were statistically significant (*p* < 0.0001).

In Figure 1d, FTIR analysis further verified the presence of BSA on the surface of the CuO nanoparticles. The FTIR spectrum of BSA@CuO-NPs showed characteristic amide I (around 1650 cm^−1^) and amide II (around 1540 cm^−1^) bands, indicating the protein’s secondary structures [29]. Additionally, distinct Cu-O stretching vibrations appeared in the region of 530 to 590 cm^−1^, which were absent in the spectrum of pure BSA. These spectral features, which were either missing or attenuated in the spectra of bare CuO and free BSA, confirm that BSA was attached to the nanoparticle surface through covalent (amide linkage via EDC/NHS) and/or non-covalent stabilization mechanisms [27,28].

Overall, the findings confirm the successful synthesis and BSA coating of CuO nanoparticles, resulting in BSA@CuO-NPs with enhanced stability, uniform dispersion, and functional surface properties.

### 3.2. Metabolic Activity and IC20 Determination of BSA@CuO-NPs in U87-MG Cells

**Figure 2:**
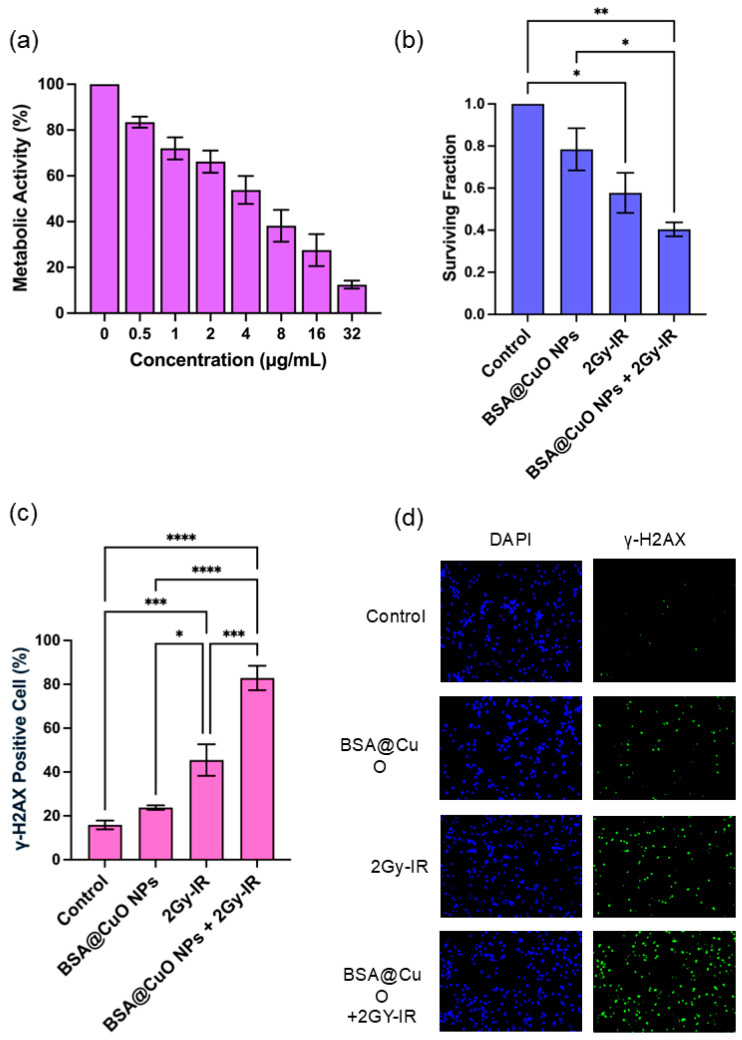
Cytotoxic and genotoxic effects of BSA@CuO-NPs on U87-MG cells. (**a**) The MTT assay was used to assess metabolic activity after 24 h of exposure. (**b**) Clonogenic survival of U87-MG cells treated with BSA@CuO-NPs at IC20 concentration, 2 Gy X-ray irradiation, or their combination. (**c**) Percentage of γ-H2AX-positive cells, an early marker of DNA double-strand breaks, measured after treatment. BSA@CuO-NPs at IC20 or 2 Gy X-ray alone increased γ-H2AX levels compared to control, while combined treatment caused a synergistic increase. (**d**) Immunofluorescence images of DAPI (blue) and γ-H2AX foci (green) are shown. All data represent mean ± SEM from at least three independent experiments. Statistical significance is indicated by * *p* < 0.05, ** *p* < 0.01, *** *p* < 0.001, and **** *p* < 0.0001.

To evaluate the impact of BSA@CuO-NPs on the metabolic activity of U87-MG cells, the cells were subjected to increasing concentrations of BSA@CuO-NPs (0–32 µg/mL) for 24 h. Typically, a concentration–response plot reveals that metabolic activity remains elevated at lower nanoparticle concentrations and diminishes progressively as the concentration rises, resulting in a characteristic dose–response curve. As depicted in Figure 2a, the MTT assay indicated a concentration-dependent reduction in cell viability, decreasing from 83.5 ± 5.9% at 0.5 µg/mL to 12.5 ± 4.3% at 32 µg/mL, relative to untreated controls. Nonlinear regression analysis of the dose–response data, conducted using GraphPad Prism, demonstrated a strong correlation (R^2^ = 0.838). The IC_20_, representing the concentration that induces a 20% reduction in viability, was approximately 0.837 µg/mL. This sublethal concentration was selected for subsequent combination experiments with ionizing radiation. The data shown in Figure 2a represent the mean ± SEM from three independent experiments conducted in triplicate, confirming the reproducibility of the observed dose–response relationship.

### 3.3. Radiosensitization Potential of BSA@CuO-NPs Assessed by Clonogenic Survival

The clonogenic survival assay was used to evaluate how BSA@CuO-NPs sensitize U87-MG cells to radiation. As shown in Figure 2b, treating cells with BSA@CuO-NPs at the IC20 alone resulted in a slight reduction in survival (SF = 0.78 ± 0.17) compared to the controls (SF = 1). Exposure to 2 Gy X-ray alone reduced survival to a greater extent (SF = 0.58 ± 0.16). Importantly, combining BSA@CuO-NPs with 2 Gy radiation resulted in the most significant decrease in clonogenicity (SF = 0.40 ± 0.06), which was significantly lower than that of either treatment alone (*p* < 0.01). These findings suggest a synergistic effect, with BSA@CuO-NPs enhancing the radiation-induced suppression of colony formation in U87-MG cells.

### 3.4. BSA@CuO-NPs Enhance Radiation-Induced DNA Damage in U87-MG Cells

To assess the DNA double-strand break (DSB) formation after treatment, γ-H2AX immunofluorescence staining was performed. The percentage of γ-H2AX-positive cells and the corresponding representative immunofluorescence images are presented, as shown in Figure 2c,d, respectively. In untreated control cells, γ-H2AX levels were low, averaging 15.8 ± 3.9%, indicating minimal DNA damage. Treatment with BSA@CuO-NPs alone at an IC20 concentration slightly increased γ-H2AX positivity to 22.8 ± 2.9%, suggesting some nanoparticle-induced genotoxic stress. Exposure to 2 Gy of X-ray irradiation alone significantly increased the percentage of γ-H2AX-positive cells (46.5 ± 18.9%, *p* < 0.01 versus control), confirming notable DNA damage from ionizing radiation. Importantly, combining BSA@CuO-NPs with 2 Gy irradiation resulted in a substantial, synergistic increase in γ-H2AX-positive cells (81.4 ± 14.5%, *p* < 0.001 compared to the control), indicating the highest level of DNA damage. These findings clearly demonstrate that BSA@CuO-NPs greatly enhance radiation-induced DNA double-strand breaks in U87-MG cells, as evidenced by increased γ-H2AX expression.

### 3.5. Induction of Apoptosis and Cell Cycle Disruption Following Combined Treatment

**Figure 3:**
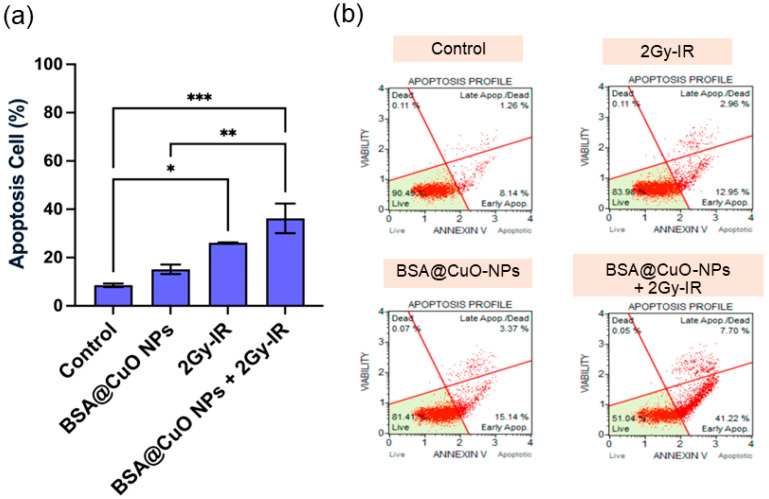
Apoptosis induction in U87-MG cells by BSA@CuO-NPs and radiation. (**a**) Percentage of apoptotic cells after treatment with BSA@CuO-NPs (0.6 µg/mL), 2 Gy irradiation, or both, assessed by Annexin V-FITC/PI staining. (**b**) Representative flow cytometry plots using Muse™ Cell Analyzers showing apoptosis profiles for each group. Data are expressed as mean ± SEM from three independent experiments. Statistical significance is indicated by * *p* < 0.05, ** *p* < 0.01, and *** *p* < 0.001.

The effects of BSA@CuO-NPs on apoptosis and cell cycle progression in U87-MG cells were assessed to understand their role in increasing radiosensitivity. In Figure 3a,b, apoptosis levels and representative Annexin V/PI flow cytometry dot plots are presented, respectively. Untreated cells had a baseline apoptosis rate of 8.53 ± 1.47%. Treatment with BSA@CuO-NPs at IC20 significantly raised apoptosis to 15.15 ± 3.92%, indicating some inherent cytotoxicity. Exposure to 2 Gy X-ray radiation alone resulted in a higher apoptosis rate of 26.16 ± 0.33%, consistent with DNA damage from radiation. Furthermore, combining BSA@CuO-NPs with irradiation resulted in a significant and statistically meaningful increase in apoptosis, reaching 36.24 ± 12.06% (*p* < 0.01 compared to control), demonstrating a synergistic effect that boosts programmed cell death.

**Figure 4:**
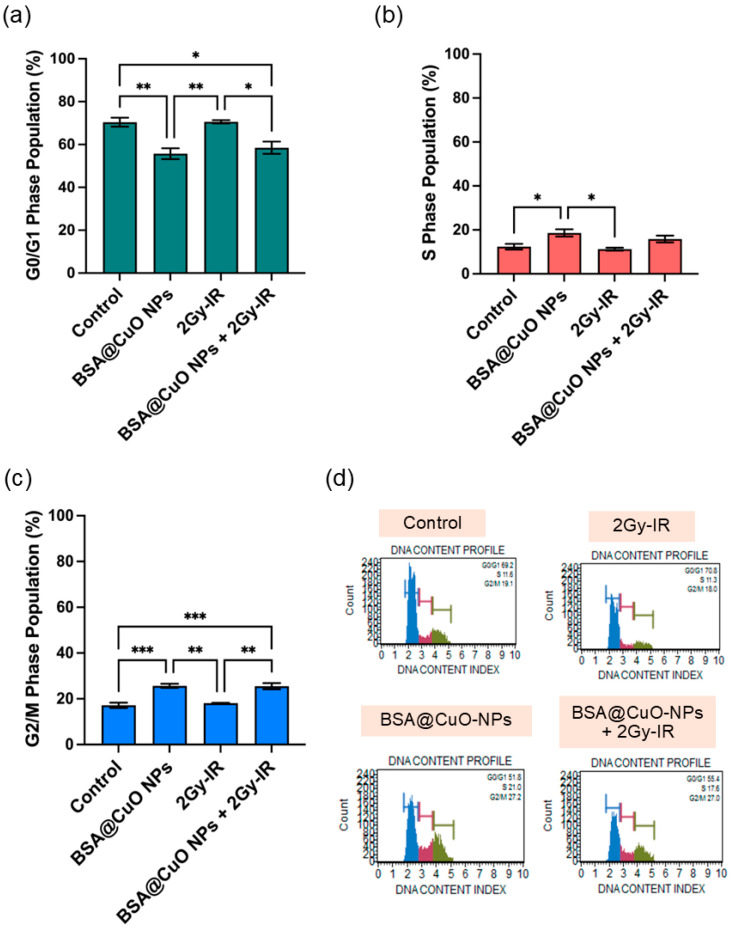
Cell-cycle distribution of U87-MG cell cycles after treatments with BSA@CuO-NPs and/or ionizing radiation treatment. (**a**–**c**) Quantification of the percentage of cells in G0/G1 (**a**), S (**b**), and G2/M (**c**) phases using Muse™ Cell Analyzer, following different conditions: control (no treatment), 2 Gy irradiation (2Gy-IR), BSA@CuO-NPs (IC20), or a combination of BSA@CuO-NPs with 2Gy-IR. (**d**) Representative DNA content histograms for each treatment, clearly marked with G0/G1 (blue), S (red), and G2/M (green) phases for better visualization. All data are presented as mean ± SEM from three independent experiments to ensure reliability. Statistical significance is indicated by * *p* < 0.05, ** *p* < 0.01, and *** *p* < 0.001, making it easy to understand the importance of the results.

To further investigate the underlying mechanism, the cell cycle distribution is analyzed in Figure 4a–d. Control cells were mostly paused in the G0/G1 phase (72.2 ± 1.8%; Figure 4a), with 11.1 ± 0.3% in the S phase (Figure 4b) and 16.8 ± 1.8% in the G2/M phase (Figure 4c). BSA@CuO-NPs alone decreased the G0/G1 population to 55.7 ± 5.1% and increased cells in the S phase (19.5 ± 8.4%) and G2/M (25.8 ± 1.3%), indicating replication stress and disruption of mitosis. Radiation alone caused G0/G1 accumulation (73.7 ± 2.9%) and reduced the S phase (7.8 ± 2.7%), with a slight increase in G2/M (18.8 ± 1.8%). The combined treatment reduced the G0/G1 population, significantly decreased S-phase cells (10.6 ± 6.8%), and increased G2/M arrest (24.9 ± 2.7%), suggesting radiosensitization due to impaired DNA repair during mitosis. Representative histograms of DNA content profiles for each treatment condition are shown in Figure 4d.

## 4. Discussion

The successful synthesis of BSA@CuO-NPs utilized EDC/NHS-mediated carbodiimide chemistry, which enabled the covalent attachment of BSA to the nanoparticle surface. This method not only improves biocompatibility but also promotes the better dispersion of nanoparticles, thereby minimizing nonspecific aggregation in biological settings [30]. BSA coating on nanoparticles plays a crucial role in enhancing their therapeutic properties, particularly in cancer treatment. BSA provides steric and electrostatic stabilization, enhances biocompatibility, reduces cytotoxic effects, and facilitates cellular uptake through interactions with albumin-binding receptors that are often upregulated in tumor cells [31,32]. DLS measurements showed a significant reduction in hydrodynamic diameter and PDI after BSA coating, consistent with prior studies indicating that protein layers decrease aggregation and enhance colloidal stability through steric and electrostatic effects [33,34]. Additionally, the shift toward a more negative zeta potential further supports this stabilization, aligning with another study demonstrating a potential role of albumin as an effective nanoparticle surface modifier [35].

SEM morphological assessment verified the nanoscale size and protein surface coating of the synthesized BSA@CuO-NPs. EDS elemental mapping confirmed nitrogen and phosphorus, consistent with the chemical composition of BSA, indicating successful immobilization on the CuO surface. These results are significant because surface functionalization affects colloidal stability and plays a crucial role in nanoparticle–cell interactions, including uptake and biodistribution within the tumor microenvironment [36]. Moreover, FTIR analysis supported these findings by displaying the characteristic amide I and II bands, indicating that the secondary structure of the protein was preserved following its binding to the nanoparticle surface. This observation aligns with prior studies, which have shown that BSA interacts with CuO nanoparticles through non-covalent hydrogen bonding and electrostatic interactions [28]. In addition, covalent amide linkages generated via EDC/NHS chemistry are known to provide irreversible and robust stabilization of protein–oxide nanoconjugates [27]. Taken together, these results suggest that the BSA coating of CuO-NPs is stabilized by a combination of covalent and non-covalent mechanisms, ensuring colloidal stability and maintaining protein integrity.

The MTT assay revealed a concentration-dependent decline in U87-MG cell viability following treatment with BSA@CuO-NPs. The calculated IC20 of 0.837 µg/mL represents a sublethal dose suitable for combination with ionizing radiation, ensuring that the observed effects on radiosensitization are not confounded by excessive cytotoxicity. This dosing approach is consistent with current nanoradiotherapy principles, focusing on biocompatible nanomaterials that minimize toxicity while boosting the therapeutic effects of radiation [37]. The mild cytotoxic response observed may be attributed to CuO-induced oxidative stress and protein interactions, consistent with established mechanisms of copper-based nanoparticle toxicity [38]. This subtoxic concentration window supports the rationale for evaluating BSA@CuO-NPs as promising radiosensitizers.

Clonogenic survival assays are still considered the gold standard for assessing radiosensitization [39]. The decrease in surviving fraction after treatment with BSA@CuO-NPs combined with 2 Gy irradiation, as shown in Figure 2b, indicates a synergistic effect between the nanoparticles and radiation. This increase in effectiveness likely results from multiple mechanisms, including increased oxidative stress, interference with DNA repair processes, and radiation-induced effects on endocytosis [6,40,41]. Similar trends have been observed with other metal-based nanoparticles, such as TiO_2_; however, the redox-active nature of CuO may contribute to a higher generation of reactive oxygen species (ROS), thereby intensifying DNA damage and reducing the clonogenic potential [42,43]. This mechanistic advantage, combined with biocompatible surface functionalization, highlights BSA@CuO-NPs as promising radiosensitizing agents for enhancing the efficacy of radiotherapy in GBM treatment.

γ-H2AX foci formation is a well-established biomarker for radiation-induced DNA double-strand breaks (DSBs). In this study, instead of quantifying discrete foci per nucleus, the percentage of γ-H2AX-positive cells was evaluated, a method commonly employed in nanotoxicology when foci overlap or nanoparticle-associated autofluorescence may confound accurate foci counting [44,45,46]. As shown in Figure 2c, γ-H2AX-positive U87-MG cells increased significantly to 81.4% after combined treatment with BSA@CuO-NPs and 2 Gy X-ray irradiation, reflecting extensive DNA damage. In comparison, BSA@CuO-NPs alone caused only a slight increase, indicating low-level genotoxic stress likely due to CuO-induced oxidative mechanisms. The notable rise in the combination group suggests a synergistic effect, possibly driven by increased ROS production and suppression of DNA repair pathways. This result highlights the stronger genotoxic response triggered by BSA@CuO-NPs in combination with ionizing radiation, supporting their potential as effective radiosensitizers for glioblastoma therapy.

The combined treatment significantly increased the number of apoptotic cells and affected cell cycle progression. As illustrated in Figure 3 and Figure 4, these findings support the mechanisms of radiosensitization by demonstrating both cell cycle arrest and induction of apoptosis. Specifically, arrest at the G2/M phase is particularly crucial because cells in this stage are highly radiosensitive due to their reduced DNA repair capacity and condensed chromatin structure [47]. The accumulation of cells in the G2/M phase following combined treatment indicates activation of cell cycle checkpoints in response to persistent DNA damage, which is concurrently associated with a significant increase in apoptotic cell death [48,49]. In this study, BSA@CuO-NPs further enhanced the sensitivity of U87-MG cells to ionizing radiation and promoted apoptosis by activating downstream signaling pathways. These observations are consistent with previous reports showing that nanoparticle surface modification can augment radiation-induced apoptosis [6]. These results are consistent with previous studies demonstrating that CuO nanoparticles can promote radiosensitization through oxidative stress and apoptotic signaling. Shafagh et al. reported that CuO nanoparticles increased ROS generation, modulated pro- and anti-apoptotic gene expression (p53, Bax, Bcl-2), and induced apoptosis in leukemia cells via mitochondrial pathways [50]. Similarly, Chakraborty et al. showed that CuO nanoparticles can act as a copper ion reservoir, leading to sustained oxidative stress and apoptosis when co-delivered with Elesclomol in lung cancer cells [51]. Together with our findings, these reports highlight the dual ability of BSA@CuO-NPs to disrupt cell cycle progression and trigger apoptotic signaling, underscoring their promise as potent radiosensitizers, particularly in treatment-resistant glioblastoma.

Despite the promising findings, several limitations must be acknowledged. Initially, the study focused only on a single glioblastoma cell line (U87-MG), which might not fully represent the biological diversity or varying radiosensitivity in GBM. Future research should incorporate multiple glioblastoma models, such as patient-derived and inherently radioresistant cell lines, to confirm whether the radiosensitizing effects of BSA@CuO-NPs under ionizing radiation conditions are broadly applicable to radiation therapy. Second, no non-malignant glial or neural cell line was used for comparison, precluding an assessment of the nanoparticles’ selective cytotoxicity and safety profile. Third, although the combined treatment with BSA@CuO-NPs and 2 Gy radiation enhanced DNA damage, apoptosis, and G2/M arrest, the underlying mechanisms, such as ROS generation and mitochondrial dysfunction, were not directly investigated. Future work should include mechanistic and in vivo studies to support clinical translation.

## 5. Conclusions

This study reports the successful creation of BSA@CuO-NPs via EDC/NHS-mediated carbodiimide chemistry, producing a stable, protein-coated nanostructure with improved physicochemical traits. In vitro tests using U87-MG cells showed that BSA@CuO-NPs are tolerated at sublethal doses but significantly enhance the effects of ionizing radiation. The combination treatment drastically decreased clonogenic survival, increased apoptosis, and caused strong G2/M cell cycle arrest. Notably, γ-H2AX analysis confirmed that BSA@CuO-NPs greatly enhance radiation-induced DNA double-strand breaks, highlighting their promising radiosensitizing ability in U87-MG cells. These results suggest that BSA@CuO-NPs are effective radiosensitizers, promoting DNA damage and cell cycle disruption to improve the radiation response. However, this remains a preclinical proof-of-concept. While promising, these findings need validation in additional glioblastoma models, mechanistic studies, and in vivo systems before clinical application. The stability of BSA@CuO-NPs in physiological fluids may support their development as future therapeutics.

## Data Availability

The data that support the findings of this study are available from the corresponding author upon reasonable request.

## Supplementary Materials

The following supporting information can be downloaded at: https://www.mdpi.com/article/10.3390/nano15171376/s1, Figure S1. SEM and EDS characterization of BSA@CuO-NPs and bare CuO-NPs. (a) SEM image of BSA@CuO-NPs shows a more compact and uniform particle surface morphology compared with bare CuO-NPs. (b) EDS spectrum of BSA@CuO-NPs confirms the presence of Cu and O, together with additional peaks of C, N, P, S, and Cl derived from the BSA coating. (c) The SEM image of bare CuO-NPs displays an irregular particle morphology with heterogeneous surface texture. (d) EDS spectrum of bare CuO-NPs shows predominantly Cu and O peaks with minor background elements, consistent with uncoated nanoparticles. Collectively, these results confirm successful BSA surface functionalization, providing distinctive protein-associated signals absent in bare CuO-NPs and supporting improved colloidal and surface properties.

## References

[1] Cella E., Bosio A., Lombardi G. (2024). New Insights into Glioblastoma. Int. J. Mol. Sci..

[2] Korbecki J., Kojder K., Grochans S., Cybulska A.M., Simi D. (2022). Epidemiology of Glioblastoma Multiforme—Literature Review. Cancers.

[3] Abedi A.A., Grunnet K., Christensen I.J., Michaelsen S.R., Muhic A., Møller S., Hasselbalch B., Poulsen H.S., Urup T. (2021). A Prognostic Model for Glioblastoma Patients Treated With Standard Therapy Based on a Prospective Cohort of Consecutive Non-Selected Patients From a Single Institution. Front. Oncol..

[4] Osuka S. (2022). Targeting adaptive radioresistance in glioblastoma. Neuro-oncology.

[5] Vilar J.B., Christmann M., Tomicic M.T. (2022). Alterations in Molecular Profiles Affecting Glioblastoma Resistance to Radiochemotherapy: Where Does the Good Go?. Cancers.

[6] Jiang Y.W., Gao G., Jia H.R., Zhang X., Zhao J., Ma N., Liu J.B., Liu P., Wu F.G. (2019). Copper Oxide Nanoparticles Induce Enhanced Radiosensitizing Effect via Destructive Autophagy. ACS Biomater. Sci. Eng..

[7] Jackson N., Cecchi D., Beckham W., Chithrani D.B. (2024). Application of High-Z Nanoparticles to Enhance Current Radiotherapy Treatment. Molecules.

[8] He M., Chen S., Yu H., Fan X., Wu H., Wang Y., Wang H., Yin X. (2025). Advances in Nanoparticle-Based Radiotherapy for Cancer Treatment. iScience.

[9] Srinivas U.S., Tan B.W.Q., Vellayappan B.A., Jeyasekharan A.D. (2019). ROS and the DNA Damage Response in Cancer. Redox Biol..

[10] Xiao S., Wang X., Chen B., Mu M., Han B., Chen N., Guo G. (2024). Enhancing Tumor Radiotherapy Sensitivity through Metal Nanomaterials: A Comprehensive Review. Malig. Spectr..

[11] Choi J., Kim G., Cho S.B., Im H.J. (2020). Radiosensitizing High-Z Metal Nanoparticles for Enhanced Radiotherapy of Glioblastoma Multiforme. J. Nanobiotechnol..

[12] Shen H., Huang H., Jiang Z. (2023). Nanoparticle-Based Radiosensitization Strategies for Improving Radiation Therapy. Front. Pharmacol..

[13] Dang Y., Guan J. (2020). Nanoparticle-Based Drug Delivery Systems for Cancer Therapy. Smart Mater. Med..

[14] Song Y., Tan K.B., Zhou S.F., Zhan G. (2024). Biocompatible Copper-Based Nanocomposites for Combined Cancer Therapy. ACS Biomater. Sci. Eng..

[15] Wei Q., Pan Y., Zhang Z., Yan S., Li Z. (2024). Copper-Based Nanomaterials for Biomedical Applications. Chem. Eng. J..

[16] Tsymbal S., Li G., Agadzhanian N., Sun Y., Zhang J., Dukhinova M., Fedorov V., Shevtsov M. (2022). Recent Advances in Copper-Based Organic Complexes and Nanoparticles for Tumor Theranostics. Molecules.

[17] Sarfraz S., Javed A., Sharif Mughal S., Bashir M., Rehman A., Parveen S., Khushi A., Kamran Khan M. (2020). Copper Oxide Nanoparticles: Reactive Oxygen Species Generation and Biomedical Applications. Int. J. Comput. Theor. Chem..

[18] Chaudhry M.A., Omaruddin R.A. (2012). Transcriptional Changes of Mitochondrial Genes in Irradiated Cells Proficient or Deficient in P53. J. Genet..

[19] Sanità G., Carrese B., Lamberti A. (2020). Nanoparticle Surface Functionalization: How to Improve Biocompatibility and Cellular Internalization. Front. Mol. Biosci..

[20] Lieberwirth I., Kokkinopoulou M., Han S., Simon J., Landfester K., Mailänder V. (2018). The Role of the Protein Corona in the Uptake Process of Nanoparticles. Microsc. Microanal..

[21] Wang J., Zhang B. (2017). Bovine Serum Albumin as a Versatile Platform for Cancer Imaging and Therapy. Curr. Med. Chem..

[22] Loureiro A., Abreu A.S., Sárria M.P., Figueiredo M.C.O., Saraiva L.M., Bernardes G.J.L., Gomes A.C., Cavaco-Paulo A. (2015). Functionalized Protein Nanoemulsions by Incorporation of Chemically Modified BSA. RSC Adv..

[23] Chaiwaree S., Prapan A., Suwannasom N., Laporte T., Neumann T., Pruß A., Georgieva R., Bäumler H. (2020). Doxorubicin–Loaded Human Serum Albumin Submicron Particles: Preparation, Characterization and in Vitro Cellular Uptake. Pharmaceutics.

[24] Atloo T., Mohammadkhani R., Mohammadi A., Zaboli K.A., Kaboli S., Rahimi H., Nosrati H., Danafar H. (2022). The Bovine Serum Albumin Coated Copper Oxide Nanoparticle for Curcumin Delivery in Biological Environment: In-Vitro Drug Release. J. Polym. Environ..

[25] Lu V.M., Crawshay-Williams F., White B., Elliot A., Hill M.A., Townley H.E. (2019). Cytotoxicity, Dose-Enhancement and Radiosensitization of Glioblastoma Cells with Rare Earth Nanoparticles. Artif. Cells Nanomed. Biotechnol..

[26] Schneider C.A., Rasband W.S., Eliceiri K.W. (2012). NIH Image to ImageJ: 25 Years of Image Analysis. Nat. Methods.

[27] Keleştemur S., Altunbek M., Culha M. (2017). Influence of EDC/NHS Coupling Chemistry on Stability and Cytotoxicity of ZnO Nanoparticles Modified with Proteins. Appl. Surf. Sci..

[28] Esfandfar P., Falahati M., Saboury A.A. (2016). Spectroscopic Studies of Interaction between CuO Nanoparticles and Bovine Serum Albumin. J. Biomol. Struct. Dyn..

[29] Miller L.M., Bourassa M.W., Smith R.J. (2013). FTIR Spectroscopic Imaging of Protein Aggregation in Living Cells. Biochim. Biophys. Acta..

[30] Ahmad Z., Shepherd J.H., Shepherd D.V., Ghose S., Kew S.J., Cameron R.E., Best S.M., Brooks R.A., Wardale J., Rushton N. (2015). Effect of 1-Ethyl-3-(3-Dimethylaminopropyl) Carbodiimide and N-Hydroxysuccinimide Concentrations on the Mechanical and Biological Characteristics of Cross-Linked Collagen Fibres for Tendon Repair. Regen. Biomater..

[31] Aires A., Ocampo S.M., Cabrera D., de la Cueva L., Salas G., Teran F.J., Cortajarena A.L. (2015). BSA-Coated Magnetic Nanoparticles for Improved Therapeutic Properties. J. Mater. Chem. B.

[32] Xie L., Tong W., Yu D., Xu J., Li J., Gao C. (2012). Bovine Serum Albumin Nanoparticles Modified with Multilayers and Aptamers for PH-Responsive and Targeted Anti-Cancer Drug Delivery. J. Mater. Chem..

[33] Tirado-Miranda M., Schmitt A., Callejas-Fernández J., Fernández-Barbero A. (2003). The Aggregation Behaviour of Protein-Coated Particles: A Light Scattering Study. Eur. Biophys. J..

[34] Lee H. (2024). Hydrodynamics and Aggregation of Nanoparticles with Protein Corona: Effects of Protein Concentration and Ionic Strength. Small.

[35] Sharma I., Sharaf M.G., Pawar A., Milley A., Unsworth L.D. (2024). Hemocompatibility of Albumin-Modified Magnetic Nanoparticles. Int. J. Mol. Sci..

[36] Mattix B., Moore T., Uvarov O., Pollard S., O’Donnell L., Park K., Horne D., Dhulekar J., Reese L., Nguyen D. (2013). Effects of Polymeric Nanoparticle Surface Properties on Interaction with Brain Tumor Environment. Nano Life.

[37] Varzandeh M., Sabouri L., Mansouri V., Gharibshahian M., Beheshtizadeh N., Hamblin M.R., Rezaei N. (2023). Application of Nano-Radiosensitizers in Combination Cancer Therapy. Bioeng. Transl. Med..

[38] Gupta G., Cappellini F., Farcal L., Gornati R., Bernardini G., Fadeel B. (2022). Copper Oxide Nanoparticles Trigger Macrophage Cell Death with Misfolding of Cu/Zn Superoxide Dismutase 1 (SOD1). Part. Fibre Toxicol..

[39] Matsui T., Nuryadi E., Komatsu S., Hirota Y., Shibata A., Oike T., Nakano T. (2019). Robustness of Clonogenic Assays as a Biomarker for Cancer Cell Radiosensitivity. Int. J. Mol. Sci..

[40] Yi X., Chen L., Chen J., Maiti D., Chai Z., Liu Z., Yang K. (2018). Biomimetic Copper Sulfide for Chemo-Radiotherapy: Enhanced Uptake and Reduced Efflux of Nanoparticles for Tumor Cells under Ionizing Radiation. Adv. Funct. Mater..

[41] Al Zaki A., Cormode D., Tsourkas A., Dorsey J.F. (2017). Increasing the Therapeutic Efficacy of Radiotherapy Using Nanoparticles. Cancer Drug Discovery and Development.

[42] Angelé-Martínez C., Nguyen K.V.T., Ameer F.S., Anker J.N., Brumaghim J.L. (2017). Reactive Oxygen Species Generation by Copper(II) Oxide Nanoparticles Determined by DNA Damage Assays and EPR Spectroscopy. Nanotoxicology.

[43] Karlsson H.L., Cronholm P., Gustafsson J., Möller L., Mo L. (2008). Copper Oxide Nanoparticles Are Highly Toxic A Comparison between Metal Oxide Nanoparticles and Carbon Nanotubes—Chemical Research in Toxicology (ACS Publications). Chem. Res. Toxicol..

[44] Mah L.J., Vasireddy R.S., Tang M.M., Georgiadis G.T., El-Osta A., Karagiannis T.C. (2010). Quantification of ΓH2AX Foci in Response to Ionising Radiation. J. Vis. Exp..

[45] Szumiel I. (2007). Analysis of DNA Double-Strand Breaks by Means of γ-H2AX Foci. Chromosomal Alterations: Methods, Results and Importance in Human Health.

[46] Böcker W., Iliakis G. (2006). Computational Methods for Analysis of Foci: Validation for Radiation-Induced γ-H2AX Foci in Human Cells. Radiat. Res..

[47] Pawlik T.M., Keyomarsi K. (2004). Role of Cell Cycle in Mediating Sensitivity to Radiotherapy. Int. J. Radiat. Oncol. Biol. Phys..

[48] Wu K.M., Chi C.W., Lai J.C.Y., Chen Y.J., Kou Y.R. (2020). TLC388 Induces DNA Damage and G2 Phase Cell Cycle Arrest in Human Non-Small Cell Lung Cancer Cells. Cancer Control.

[49] Gazdova M., Michalkova R., Kello M., Vilkova M., Kudlickova Z., Baloghova J., Mirossay L., Mojzis J. (2022). Chalcone-Acridine Hybrid Suppresses Melanoma Cell Progression via G2/M Cell Cycle Arrest, DNA Damage, Apoptosis, and Modulation of MAP Kinases Activity. Int. J. Mol. Sci..

[50] Shafagh M., Rahmani F., Delirezh N. (2015). CuO Nanoparticles Induce Cytotoxicity and Apoptosis in Human K562 Cancer Cell Line via Mitochondrial Pathway, through Reactive Oxygen Species and P53. Iran. J. Basic Med. Sci..

[51] Chakraborty S., Prakash P., Shah J., Mayya C., Singh S., Ranganathan R., Soppina V., Jones E.V., Misra S.K. (2022). CuO Nanoparticles as Copper-Ion Reservoirs for Elesclomol-Mediated Intracellular Oxidative Stress: Implications for Anticancer Therapies. ACS Appl. Nano Mater..

