# Supplementary material for: Albumin-Coated Copper Oxide Nanoparticles for Radiosensitization of Human Glioblastoma Cells Under Clinically Relevant X-Ray Irradiation
Source: Nanomaterials (Basel). 2025 Sep 5;15(17):1376. doi: 10.3390/nano15171376 (PMC12430620; doi:10.3390/nano15171376)
Supplement: Supplementary file 1 [file nanomaterials-15-01376-s001.zip › nanomaterials-3839941-supplementary.pdf]

Albumin-Coated Copper Oxide Nanoparticles for Radiosensitization of Human Glioblastoma Cells Under Clinically Relevant X-Ray Irradiation

Chanyatip Suwannasing [1], Nittiya Suwannasom[2], Pattawat Iamcharoen[1], Rachan Dokkham[1], Panupong Maun[1], Pitchayuth Srisai[3], Hans Bäumler [4,5], Ausanai Prapan [1,*]

[1] Department of Radiological Technology, Faculty of Allied Health Sciences, Naresuan University, Mueang District, Phitsanulok 65000, Thailand
[2] Division of Biochemistry, School of Medical Sciences, University of Phayao, Phayao, 56000, Thailand
[3] Department of Biology, Faculty of Science, Chiang Mai University, Chiang Mai, 50200, Thailand
[4] Institute of Transfusion Medicine, Charité-Universitätsmedizin Berlin, 10117 Berlin, Germany
[5] Department of Pharmaceutical Technology and Biotechnology, Faculty of Pharmacy, Payap University, Chiang Mai, Mueang District, Chiang Mai 50000, Thailand

* Correspondence: Ausanai Prapan

Supporting Information

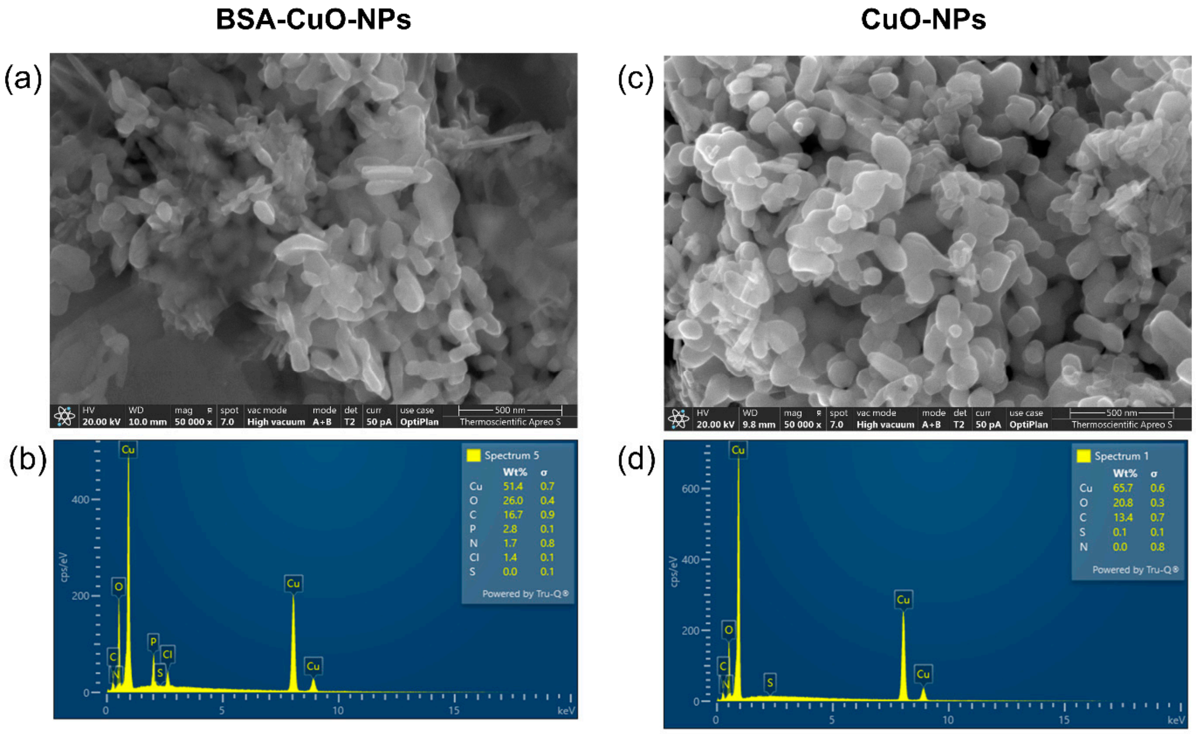

Figure S1. SEM and EDS characterization of BSA@CuO-NPs and bare CuO-NPs.

(a) SEM image of BSA@CuO-NPs shows a more compact and uniform particle surface morphology compared with bare CuO-NPs. (b) EDS spectrum of BSA@CuO-NPs confirms the presence of Cu and O, together with additional peaks of C, N, P, S, and Cl derived from the BSA coating. (c) The SEM image of bare CuO-NPs displays an irregular particle morphology with heterogeneous surface texture. (d) EDS spectrum of bare CuO-NPs shows predominantly Cu and O peaks with minor background elements, consistent with uncoated nanoparticles. Collectively, these results confirm successful BSA surface functionalization, providing distinctive protein-associated signals absent in bare CuO-NPs and supporting improved colloidal and surface properties
